# Vasodilator Strain Stress Echocardiography in Suspected Coronary Microvascular Angina

**DOI:** 10.3390/jcm11030711

**Published:** 2022-01-28

**Authors:** Hugo Rodriguez-Zanella, Rosina Arbucci, Juan Francisco Fritche-Salazar, Xochitl Arely Ortiz-Leon, Domenico Tuttolomondo, Diego Haber Lowenstein, Karina Wierzbowska-Drabik, Quirino Ciampi, Jarosław D. Kasprzak, Nicola Gaibazzi, Jorge Lowenstein, Edith Liliana Posada-Martinez, Jose Antonio Arias-Godinez, Juan C. de la Fuente-Mancera, Eugenio Picano, on behalf of the Stress Echo 2020 Study Group of the Italian Society of Echocardiography and Cardiovascular Imaging (SIECVI)

**Affiliations:** 1 National Institute of Cardiology Ignacio Chavez, Mexico City 14080, Mexico; drzanella@gmail.com (H.R.-Z.); xochitlortiz6@gmail.com (X.A.O.-L.); liliposada1701@gmail.com (E.L.P.-M.); jaag1407@yahoo.com.mx (J.A.A.-G.); delafuentemancera@gmail.com (J.C.d.l.F.-M.); 2 Cardiodiagnosticos Investigaciones Medicas, Buenos Aires C1082, Argentina; rosinaarbucci@hotmail.com (R.A.); lowediego@hotmail.com (D.H.L.); lowensteinjorge@hotmail.com (J.L.); 3 Cardiology Department Parma University Hospital, 43100 Parma, Italy; d.tuttolomondo@hotmail.it (D.T.); ngaibazzi@gmail.com (N.G.); 4 I Department of Cardiology, Bieganski Hospital, Medical University, 91-347 Lodz, Poland; wierzbowska@ptkardio.pl (K.W.-D.); kasprzak@ptkardio.pl (J.D.K.); 5 Cardiology Division Fatebenefratelli Hospital, 82100 Benevento, Italy; qciampi@gmail.com; 6 CNR Institute of Clinical Physiology Biomedicine Department, 56100 Pisa, Italy; picano@ifc.cnr.it

**Keywords:** non-obstructive coronary artery stenosis, two-dimensional speckle-tracking echocardiography, stress echocardiography, coronary flow velocity reserve

## Abstract

**Background:** In patients with Ischemia and non-obstructive coronary artery stenosis (INOCA) wall motion is rarely abnormal during stress echocardiography (SE). Our aim was to determine if patients with INOCA and reduced coronary flow velocity reserve (CVFR) have altered cardiac mechanics using two-dimensional speckle-tracking echocardiography (2DSTE) during SE. **Methods:** In a prospective, multicenter, international study, we recruited 135 patients with INOCA. Overall, we performed high dose (0.84 mg/kg) dipyridamole SE with combined assessment of CVFR and 2DSTE. The population was divided in patients with normal CVFR (>2, group 1, *n* = 95) and abnormal CVFR (≤2, group 2, *n* = 35). Clinical and 2DSTE parameters were compared between groups. **Results:** Feasibility was high for CFVR (98%) and 2DSTE (97%). A total of 130 patients (mean age 63 ± 12 years, 67 women) had complete flow and strain data. The two groups showed similar 2DSTE values at rest. At peak SE, Group 1 patients showed lower global longitudinal strain (*p* < 0.007), higher mechanical dispersion (*p* < 0.0005), lower endocardial (*p* < 0.001), and epicardial (*p* < 0.0002) layer specific strain. **Conclusions:** In patients with INOCA, vasodilator SE with simultaneous assessment of CFVR and strain is highly feasible. Coronary microvascular dysfunction is accompanied by an impairment of global and layer-specific deformation indices during stress.

## 1. Introduction

Patients with angina and non-obstructive coronary artery stenosis (INOCA) are increasingly recognized in clinical practice. These patients are at higher risk of major adverse cardiovascular events [1,2,3], and experience recurrent symptoms. Microvascular angina is the most common mechanism of ischemia in patients with INOCA [4,5].

Vasodilator stress echocardiography allows to measure non-invasively coronary flow velocity reserve (CFVR) [6,7] and is recommended by current guidelines in patients with suspected microvascular angina [8]. Despite important abnormalities in patients with INOCA, stress echocardiography rarely demonstrates regional wall motion (RWM) abnormalities [7]. Two-dimensional speckle-tracking echocardiography (2DSTE) has been shown to be useful to detect subclinical left ventricular (LV) dysfunction and dyssynchrony in different diseases. Accordingly, we aimed to determine if microvascular angina, assessed by CFVR, might cause changes in myocardial mechanics during vasodilator stress echocardiography reflecting subclinical LV dysfunction and dyssynchrony.

## 2. Materials and Methods

### 2.1. Study Population

In this prospective study we enrolled patients recruited by three laboratories in three countries (Mexico, Argentina, and Poland). The inclusion criteria were: (1) age >18 years; (2) referral for chest pain or dyspnea as the primary symptom and chest pain and absence of obstructive coronary artery disease (CAD) defined as lesions <49% by either invasive coronary angiography (ICA) or coronary computed tomography angiography (CCTA); (3) normal wall motion during high dose (0.84 mg/kg) dipyridamole stress echocardiography (SE); (4) no severe valvular heart disease or congenital heart disease; (5) adequate acoustic window for 2DSTE analysis at rest; (6) adequate images to assess coronary flow using transthoracic Doppler mid left anterior descending (LAD) artery interrogation at rest; (7) willingness to give informed consent for allowing scientific use of the observational data, which are respectful of privacy rights. The study protocol was approved by the institutional ethics committees as a part of the SE 2020 study (148-Comitato Etico Lazio-1, 16 July 2016; Clinical trials. Gov Identifier NCT 030). Patients studied with Philips machine in one center Parma, Italy were excluded from the study, due to inter-vendor differences for strain analysis.

Microvascular angina was defined as the presence of symptoms of ischemia in the absence of obstructive CAD (lesion <49% on CCTA performed in 64 (49%) patients or ICA performed in 66 (51%) patients), and evidence of impaired coronary microvascular function assessed by vasodilator stress echocardiography with a CFVR ≤ 2 [9]. Briefly, CFVR refers to the ratio of coronary velocity in the left anterior descending artery measured during a hyperemic stimulus divided by the coronary velocity in the left anterior descending artery at rest. In the absence of obstructive epicardial disease a reduced CVFR ≤ 2 indicates abnormal microvascular function.

### 2.2. Rest and Stress Echocardiography

All subjects underwent a comprehensive two-dimensional (2D) echocardiographic study. Studies were performed using a Vivid E9 ultrasound machine (GE Vingmed Ultrasound AS, Horten, Norway) equipped with an M5S probe. LV volumes used to calculate ejection fraction (EF) were measured by modified biplane Simpson’s method according to the American Society of Echocardiography and European Association of Cardiovascular Imaging [10].

**Figure 1:**
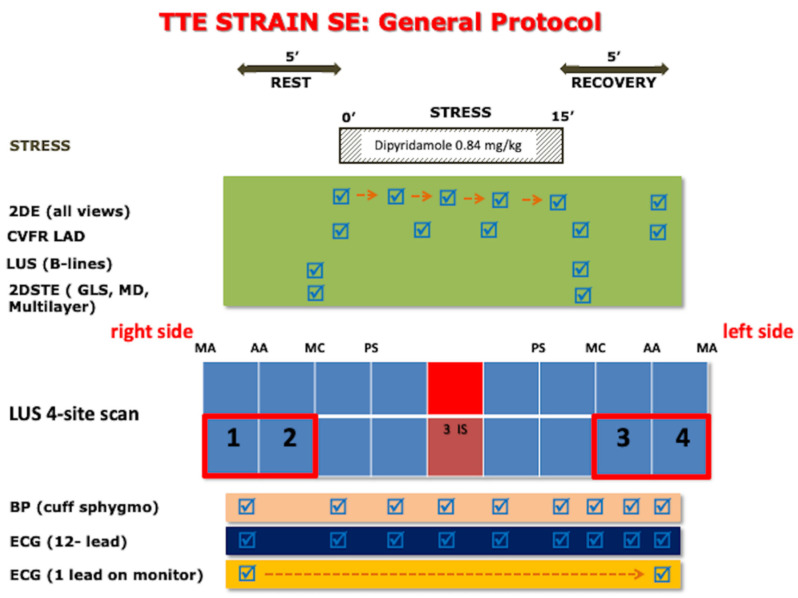
Transthoracic strain stress echo protocol. Intermittent imaging of LAD flow is performed at rest and peak stress with the same transducer used for continuous 2DE of wall motion. 2DE: two-dimensional echocardiography; 2DSTE: Two-dimensional speckle-tracking echocardiography; BP: blood pressure; CFVR: coronary flow velocity reserve; ECG: electrocardiogram; GLS: global longitudinal strain; LAD: left anterior descending artery; LUS: lung ultrasound; MD: mechanical dispersion; MA: mid-axillary; AA: anterior-axillary; MC: mid-clavicular; PS: parasternal; SE: stress echocardiography.

Patients underwent pharmacological SE according to the protocol recommended by the American Society of Echocardiography and the European Association of Cardiovascular Imaging [11,12]. We used a dipyridamole dose of 0.84 mg/kg over 6 min. The electrocardiogram was monitored continuously and blood pressure intermittently. The general transthoracic echocardiography (TTE) strain SE protocol is shown in Figure 1. Criteria for interrupting the test were severe chest pain, diagnostic ST-segment shift, excessive blood pressure increase (systolic blood pressure ≥240 mmHg, diastolic blood pressure ≥120 mmHg), dyspnea, maximal predicted heart rate, or significant arrhythmias. Echocardiographic studies included three apical views (four-chamber, two-chamber, and long-axis) optimized for global longitudinal strain (GLS) analysis. For each view, 3 consecutive heart cycles were recorded with a frame rate ranging between 50 and 80 frames/s. Data sets were stored digitally and analyzed offline. All physicians and nurses involved were trained in Basic Life Support and Advanced Cardiac Life Support.

The imaging protocol included step A for RWM analysis; step B for B-lines assessment with lung ultrasound and 4-site simplified scan [13,14]; step C for assessment of LV contractile reserve (LVCR) with EF and force (systolic blood pressure [SBP]/end-systolic volume); step D for Doppler based assessment of CFVR; and step E for electrocardiogram (EKG)-based assessment of heart rate reserve (HRR). In particular, for step D, Coronary flow in the mid-distal portion of the LAD were imaged from the low parasternal long-axis under the guidance of color Doppler flow mapping [6]. Flow velocities were measured at baseline and at peak stress. At each time point, three optimal profiles of peak diastolic Doppler flow velocities were measured, and the results were averaged. CFVR was defined as the ratio between hyperemic peak and basal peak diastolic coronary flow velocities [15]. The force-based assessment of LVCR was also calculated as the stress/rest ratio of force (defined as systolic blood pressure/end-systolic volume) [16]. All readers (one for each center) underwent a web-based training and quality control as previously described for RWM [17], B-lines [18], end-systolic volume and CFVR [19]. As a part of the quality control process of the SE 2020-CFVR subproject, the accredited readers all had ≥90% concordance with core laboratory reading on measurement of peak diastolic flow velocity in a set of 20 clips selected from 8 different laboratories. The interobserver variability was <10% and the intraclass correlation coefficient (ICC) coefficient was >90% for all accredited readers. The previously assessed intraobserver variability was <5% [15].

Using 2DSTE, GLS was obtained by averaging the measures of each segment peak systolic longitudinal strain value. Timing of aortic valve closure was selected using automatic software timing [20]. Inclusion of LV segments for analysis required the operator’s approval. Position and width adjustment of the automatically drawn region of interest (ROI) were performed when necessary. In patients with more than two inadequately tracked LV segments in one view, GLS, layer specific strain (LSS), and mechanical dispersion (MD) were not computed and were excluded from analysis. MD was defined as the standard deviation of the time from the peak of the R wave on the electrocardiogram to the peak negative strain using a 16 segment LV model. LSS calculation was performed paying attention to cover the entire myocardial wall thickness by the ROI of each segment. Subendocardial longitudinal strain (LSsubendo) and subepicardial longitudinal strain (LSsubepi) were measured on the endocardial and epicardial ROI border, respectively. Whereas the mid-myocardial, the center line of the ROI, represented the average values of the transmural wall thickness. Longitudinal strain gradient was calculated as the difference between LSsubendo and Lssubepi. All calculations of MD, LSS, and GLS were done using commercially available software package (EchoPAC version BT113, GE Vingmed Ultrasound, Horten, Norway). GLS and LSS are expressed as absolute values.

Strain reserve (SR) was calculated as the ratio of GLS in maximal hyperemia, divided by the GLS at rest.

### 2.3. Statistical Analysis

Continuous variables are expressed as mean and standard deviation (SD) or median and interquartile range (IQR), as appropriate. Differences in continuous variables between groups (CFVR ≤ 2 vs. CFVR > 2) were tested using t-test or Wilcoxon signed rank according to their distribution. For variables with sequential evaluation at rest and stress, the Student’s paired test was used. Categorical variables are expressed as absolute numbers with percentages, comparisons between them were made using Pearson’s Chi-squared or Fisher´s exact test. We considered statistically significance if *p* < 0.05. All analyses were made with Stata 14.0 (StataCorp, College Station, TX, USA). The reproducibility of strain analysis of our group is high and has been previously reported. Reproducibility analysis was performed in 20 subjects in whom LVCR was measured by a second investigator. Reproducibility was reported using ICC with a two-way mixed model for absolute agreement.

## 3. Results

Of the 500 patients screened for suspected chronic coronary syndrome, 135 patients (26%) had normal coronary arteries and suspected microvascular angina. Seventy-nine studies were performed in Mexico, 41 in Argentina, and 10 in Poland. None of the patients included had a criterion for test interruption.

Feasibility was high for CFVR (133/135, 98%) and 2DSTE (132/135, 97%). Of the resulting population of 130 patients (mean age 63 ± 12 years, 67 women) with complete flow and strain data, 35 (27%) showed abnormal (≤2.0, Group 1) and 95 normal (>2.0, Group 2) CFVR.

**Figure 2:**
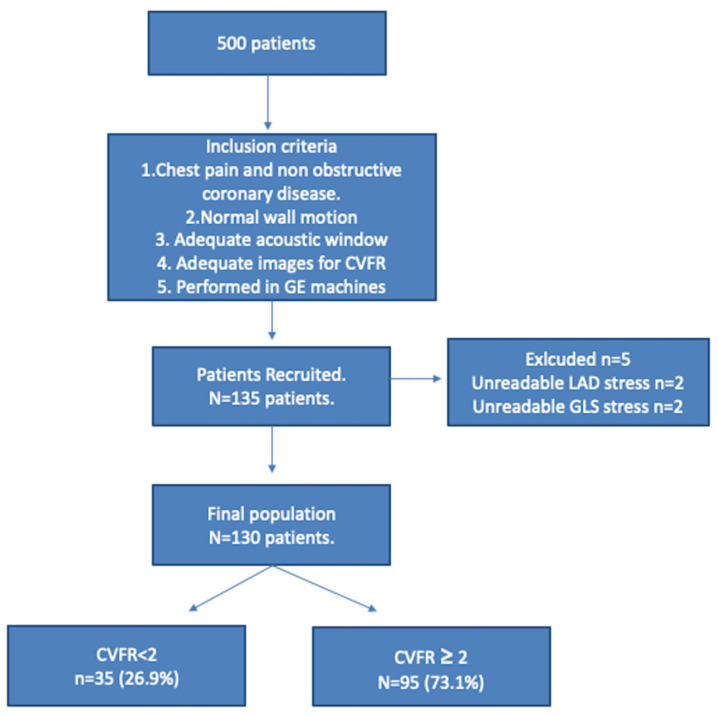
Study flow chart. CVFR: coronary flow velocity reserve; GE: General Electric.

The study flowchart is shown in Figure 2. The main clinical characteristic of the patients is presented in Table 1.

Thirty-five patients (27%) had reduced CVFR compatible with microvascular angina. Patients with microvascular angina had comparable baseline demographic characteristics and comorbidities, higher baseline systolic and diastolic blood pressure, and were more often treated with calcium channel blockers (Table 1 and Table 2). Baseline echocardiographic examination showed comparable features except for a trend for higher baseline dyssynchrony by MD in patients with microvascular angina (Table 3).

**Figure 3:**
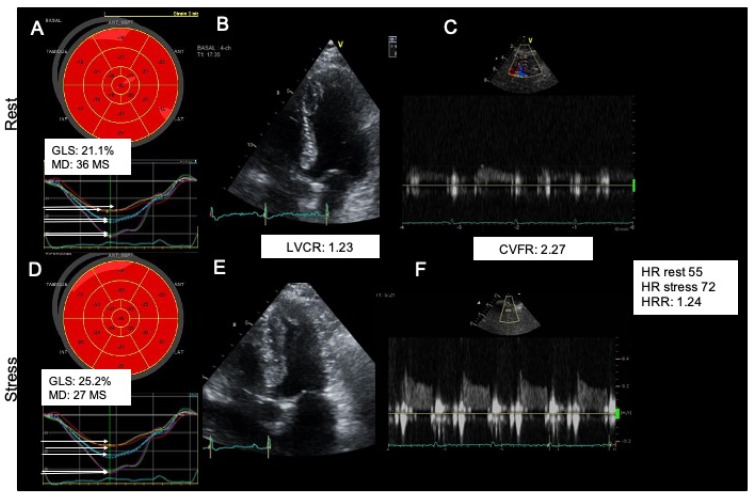
A typical normal response of a normokinetic and strained (**A** strain normal), dry (**B** step normal) not shown in the image, strong (**C** step normal), warm (**D** step normal) and fast (**E** step normal) heart. Upper panel shows rest findings: Strain (**A**), LVCR (**B**), CVFR (**C**). Lower panel shows stress findings Strain (**D**), LVCR (**E**), CVFR (**F**). GLS: global longitudinal strain, MD: mechanical dispersion, CVFR: coronary flow velocity reserve, HR: heart rate, HRR: heart rate reserve LVCR: Left ventricular contractile reserve.

**Figure 4:**
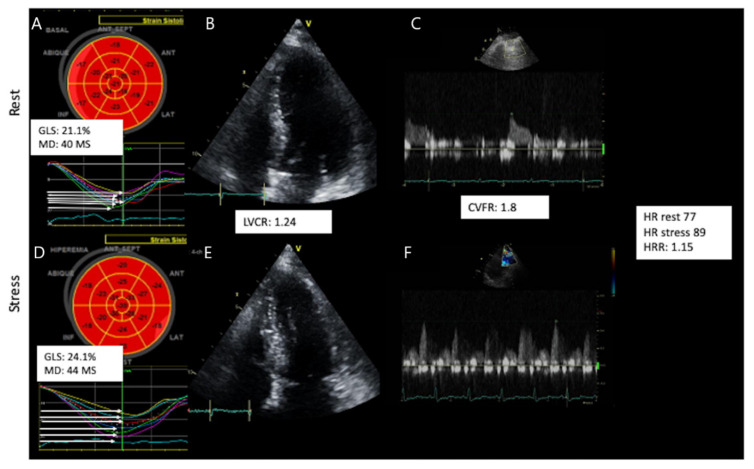
A typical abnormal response of a normokinetic and de-strained (**A** strain abnormal), dry (**B** step abnormal) not shown in the image, strong (**C** step normal), cold (**D** step abnormal) and slow (**E** step abnormal) heart. Upper panel shows rest findings: Strain (**A**), LVCR (**B**), CVFR (**C**). Lower panel shows stress findings Strain (**D**), LVCR (**E**), CVFR (**F**). GLS: global longitudinal strain, MD: mechanical dispersion, CVFR: coronary flow velocity reserve, HR: heart rate, HRR: heart rate reserve LVCR: Left ventricular contractile reserve.

Figure 3 and Figure 4 show examples of a typical response in patients of group 1 (abnormal CVFR) and group 2 (normal CVFR) and their corresponding changes in myocardial mechanics at rest and hyperemia. During vasodilator SE patients with microvascular angina had lower GLS (*p* = 0.007), higher dyssynchrony by MD (*p* = 0.0005), and lower LSS both endocardial (*p* = 0.001) and epicardial (0.0002), whereas LVCR was not different between groups; however, the strain stress-rest delta (<0.0001) and SR (<0.0001) were reduced in patients with INOCA and reduced CVFR. Patients with INOCA and reduced CVFR also had abnormal autonomic function reflected by a reduced HRR (*p* = 0.044) (Table 4).

Because synchrony and strain are central to our findings, we performed sensitivity analysis excluding patients with LBBB for the main strain and synchrony parameters. For the sensitivity analysis, GLS (*p* = 0.0013), MD (*p* = 0.0007), LV synchrony delta (*p* = 0.045), and strain stress-rest delta (*p* = 0.0001) remained significant.

### Feasibility and Reproducibility Analysis

On 2DSTE 98% of the segments could be measured. Feasibility in our study group at rest and during vasodilator stress was 97.7%. Intra- and inter-observer intraclass correlations for GLS and MD by strain echocardiography were 0.96 (95% confidence interval (CI): 0.90 to 0.98) and 0.94 (95% CI: 0.88 to 0.98), respectively, and 0.91 (95% CI: 0.76 to 0.96) and 0.87 (95% CI: 0.69 to 0.95). Our inclusion criteria required an adequate acoustic window and images for CFVR, which could explain the high feasibility. Fourteen patients could not be included due to inadequate acoustic window or images for CFVR (either inadequate window or irregular rhythm) and therefore the overall feasibility would be 87%.

## 4. Discussion

**Figure 5:**
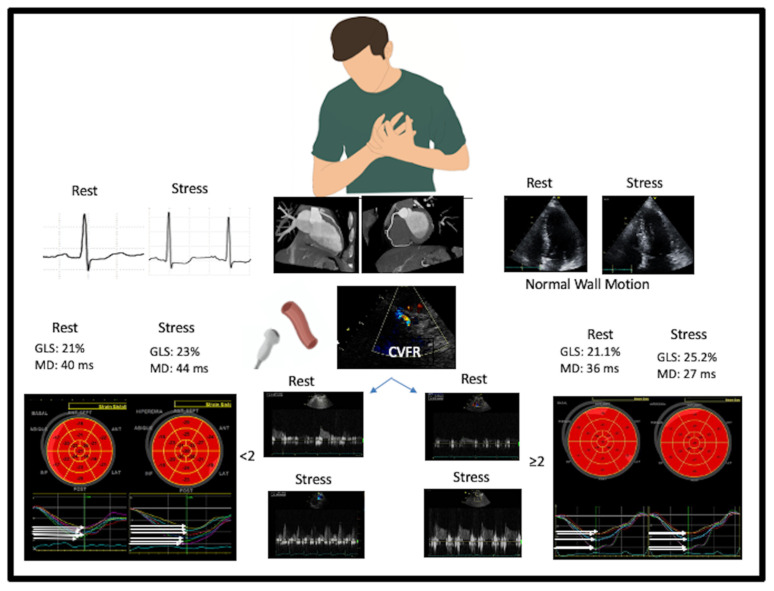
The top panel shows the clinical presentation of coronary microvascular angina with chest pain, the ST segment depression during exercise or pharmacological stress (left panel), normal coronary arteries (middle panel with MDCT), and the usual 2D pattern or SE with regional and global hyperkinetic response during SE (right panel). During SE, coronary flow velocity is assessed. This same pattern is associated with two distinct responses. Left panel: a positive response with confirmed coronary microvascular response: reduced coronary flow velocity reserve with abnormal strain and increased mechanical dyssynchrony. Right panel: a negative response with ruled-out coronary microvascular response: normal coronary flow velocity reserve with normal strain and increased mechanical synchrony. GLS: global longitudinal strain, MD: mechanical dispersion, CVFR: coronary flow velocity reserve, HR: heart rate; 2D: two-dimensional, SE: stress echocardiography.

The main findings of this study can be summarized as follows: (i) the assessment of myocardial mechanics during vasodilator stress echocardiography is highly feasible (ii) patients with INOCA and coronary microvascular dysfunction have reduced global longitudinal strain and increased dyssynchrony unmasked during stress, despite having normal wall motion. (iii) LVCR based on EF or load-independent force is not different in patients with normal or abnormal CFVR, while myocardial mechanics expressed by global and layer specific deformation indices is reduced in patients with INOCA and coronary microvascular dysfunction. These more sensitive novel parameters likely reflect myocardial dysfunction during stress, even in the absence of wall motion abnormalities. Figure 5 summarizes the main findings of our study.

INOCA patients are a heterogeneous cohort. Our findings show that a comprehensive SE is useful in order to identify different phenotypes of the disease, including coronary microvascular dysfunction mirrored by reduced CFVR, abnormal cardiac sympathetic reserve identified by blunted HRR [21], and true mechanical ischemia with strain alterations. According to John Ross Jr, “ischemia is a reduction in myocardial blood flow sufficient to cause a decrease in myocardial contraction” [22]. When ischemia does not reach a critical myocardial mass in vertical (subendocardial to subepicardial) or horizontal (across segments) direction, regional and global indices of LV function are normal, but deformation indices are impaired during stress in CAD patients [23]. Strain imaging helps to solve the paradox of a reduced CFVR with normal regional wall thickening and normal global contractile reserve, since deformation indices are impaired in these patients, and these strain indices are known to be more sensitive to ischemia than conventional indices of regional or global LV function. The finding of strain alterations despite normal wall motion corroborates the true ischemic nature of chest pain, more likely in the subset of INOCA patients with impaired CFVR and altered cardiac autonomic function.

Our study supports the evidence regarding the feasibility of CVFR measurement during stress echocardiography. Previous studies have shown good feasibility depending on the stressor, 81% for dobutamine, 80% for exercise, and 95% for vasodilators [6,24,25,26]. The overall feasibility in our study is comparable. Feasibility might be further increased by using ultrasound enhancing agents in patients with challenging acoustic windows to assess CVFR.

Myocardial deformation has been shown to be impaired in a number of diseases such as ischemic heart disease [26,27], and heart failure with reduced [28] and preserved ejection fraction [5]. However, less compelling evidence exists in patients with INOCA with microvascular dysfunction.

In a sub study of the iPower trial that included 963 women, 26% had coronary microvascular dysfunction defined as CFVR ≤ 2. Coronary microvascular dysfunction was not associated with rest LVEF and GLS. However, these patients had reduced GLS at peak hyperemia and GLS reserve measured as the delta between stress and basal GLS [29]. The observation of Michelsen et al. in women is confirmed by the present study, which included almost 50% of men. Furthermore, it has been shown in untreated hypertensive patients that impaired CVFR (<2.5) have a significant association with an abnormal response of LV systolic and diastolic function assessed by tissular doppler imaging (TDI)-derived indices during adenosine stress echocardiography, most likely reflecting an early subclinical myocardial dysfunction of the LV in these patients [30]. Our findings support the notion that INOCA with microvascular angina may cause subtle myocardial dysfunction that can be detected using 2DSTE indices only after vasodilator stress echocardiography. In our study, neither LVEF nor LVCR were different in patients with INOCA and microvascular angina, even for stress parameters, as LVCR was not different.

Cardiac autonomic function might also be impaired in patients with microvascular angina and can be assessed during vasodilator stress echocardiography using HRR. In our study, HRR was lower among patients with coronary microvascular dysfunction, supporting this notion.

The effect of stress echocardiography on LV synchrony has been sparsely studied. Previous studies using TDI to calculate strain and strain rate curves, suggested increased dyssynchrony in patients with LV dysfunction [28,31]. However, to the best of our knowledge, ours is the first study to evaluate LV dyssynchrony with speckle tracking in patients with microvascular angina. We used MD to assess LV dyssynchrony as it can be easily obtained in addition to GLS. In our population, MD is higher than the normal values previously reported [32]. Furthermore, in patients with microvascular angina MD does not decrease after stress as previously reported in healthy sedentary subjects and healthy athletes [33]. This explains the higher dyssynchrony and might be a sign of LV dysfunction.

## 5. Limitations

A single vendor machine was used in the study in the three recruiting centers, and data obtained with another vendor in a fourth center was excluded from further analysis. This may limit the generalizability of our findings. However, this was a necessary step in order to avoid the known source of bias of intervendor variability of strain data [12]. Vendor-neutral approaches are now being developed to allow across-vendors standardization of strain acquisition, analysis, and values. In addition, global longitudinal strain can be analyzed with vendor-independent systems based on artificial intelligence, which are easy to incorporate in echocardiographic data analysis.

We also used one type of stress modality, i.e., high dose dipyridamole. This choice has a methodological and clinical reason. This is the recommended modality for non-invasive assessment of CFVR in INOCA patients, to document coronary microvascular dysfunction as a specific phenotype according to the latest 2020 European Society of Cardiology guidelines in chronic coronary syndromes [8]. Furthermore, dipyridamole was the optimal stress modality to improve the feasibility and robustness of 2DSTE, since strain values are affected by changing the loading conditions, and most importantly by inadequate frame rates in the setting of tachycardia associated with stress. Dipyridamole stress is associated with a minimal increase in heart rate (on average, from 69 to 85 bpm in our population) and no change in SBP (an index of afterload) and end-diastolic volume (an index of preload). It allowed simultaneous assessment of function (step A), pulmonary congestion (step B), cardiac reserve (step C), CFVR (step D), heart rate reserve (step E), and GLS, which unmasked a functional impairment missed by RWM abnormalities. Whether this finding applies also to other more technically challenging stress modalities for assessment of CFVR and strain, such as exercise or dobutamine, remains to be established.

The higher rest SBP and diastolic blood pressure in the CFVR ≤ 2 group compared to those with CFRV > 2 indicates that hypertension and associated increased arterial stiffness may determine impairment CFVR (e.g., through microvascular compression by increased LV diastolic filling pressure) as previously reported [30,34] even after successful percutaneous coronary intervention in CAD [35].

Outcome information was not available, but a reduced CFVR identifies a higher risk subset in patients with INOCA without inducible RWM abnormalities [36]. Future studies on larger series, currently ongoing in the framework of Stress echo 2030, will establish whether CFVR, HRR, and strain-based mechanical alterations may have independent and incremental prognostic value in these patients.

## 6. Conclusions

In patients with INOCA, vasodilator stress echocardiography is a feasible and clinically valuable method to detect reduced CVFR. Patients with INOCA and reduced CVFR have altered cardiac mechanics during peak hyperemia. Reduced GLS and LSS, appear to reflect LV subclinical ischemic dysfunction during stress. Further studies should look at the value of these novel indices to assess prognosis in patients with microvascular angina.

## Figures and Tables

**Table 1:** Clinical characteristics of the population.

	General Population	CFVR ≤ 2.0	CFVR > 2.0	
	*n* = 130	*n* = 35	*n* = 95	*p*
Age (years)	63 ± 12	68 ± 11	62 ± 12	0.012
Female (%)	67 (51.4)	18 (51.4)	49 (51.6)	0.988
Height (cm)	161 ± 9	159 ± 10	161 ± 8	0.073
Weight (kg)	72.7 ± 13.8	69.6 ± 14.1	73.5 ± 13	0.505
BMI (kg/m^2^)	28.1 ± 5.2	27.7 ± 6.4	28.3 ± 4.6	0.251
BSA (m^2^)	1.79 ± 0.2	1.75 ± 0.21	1.8 ± 0.2	0.122
Indication of stress test (%)				0.298
Dyspnea as the primary symptom	12 (9.2)	5 (14.3)	7 (7.4)	
Atypical chest pain	76 (58.5)	20 (57.1)	56 (59)	
Typical chest pain	25 (19.2)	4 (11.4)	21 (22.1)	
High clinical risk	15 (11.5)	6 (17.1)	9 (9.5)	
Re-stratification	2 (1.5)	0	2 (2.1)	
LBBB (%)	8 (6.2)	2 (5.7)	6 (6.3)	0.631
Hypertension (%)	92 (70.7)	28 (80)	64 (86.4)	0.195
Diabetes (%)	41 (31.5)	12 (34.3)	29 (30.5)	0.677
Smoker (%)				0.165
Current	28 (21.5)	4 (11.4)	24 (25.3)	
Former	32 (24.6)	8 (22.8)	24 (25.3)	
Dyslipidemia (%)	60 (46.1)	13 (37.1)	47 (49.5)	0.211
Dialysis (%)	3 (2.3)	2 (5.7)	1 (1.1)	0.176
COPD (%)	13 (10)	2 (5.7)	11 (11.6)	0.512

CFVR: coronary flow velocity reserve, BMI: body mass index, BSA: body surface area, LBBB: left bundle branch block, COPD: chronic obstructive pulmonary disease.

**Table 2:** Medical treatment of the population and comparison between groups.

	General Population	CFVR < 2.0	CFVR ≥ 2.0	
	*n* = 130	*n* = 35	*n* = 95	*p*
Beta blocker (%)	59 (45.4)	14 (40)	45 (47.4)	0.454
ACE inhibitors (%)	54 (42.5)	15 (42.8)	39 (41.1)	0.853
ARA-2 inhibitors (%)	38 (29.2)	13 (37.2)	25 (26.3)	0.278
ARB´s (%)	5 (3.8)	13 (37.1)	25 (26.3)	0.722
Diuretics (%)	23 (17.7)	6 (17.1)	17 (17.9)	0.921
Digitalis (%)	1 (0.8)	0	1 (1.1)	0.542
Calcium antagonist (%)	31 (23.8)	14 (40)	17 (17.9)	0.009
Nitrate (%)	8 (6.2)	3 (8.6)	5 (5.3)	0.486
Aspirin (%)	79 (60.8)	22 (62.8)	57 (60)	0.841
Antiplatelet agent (%)	20 (15.4)	4 (11.4)	16 (16.8)	0.448
Antidiabetic drugs (%)	27 (20.8)	8 (22.9)	19 (20)	0.722
Insulin (%)	12 (9.2)	3 (8.6)	9 (9.5)	0.875
Anticoagulant (%)	12 (9.2)	2 (5.7)	10 (10.5)	0.512
Statins (%)	69 (53.1)	16 (45.7)	53 (55.8)	0.307

CFVR: coronary flow velocity reserve, ACE: angiotensin converting enzyme, ARA-2: angiotensin 2 receptor antagonist, ARBs: angiotensin receptor blockers.

**Table 3:** Clinical and echocardiographic characteristics at rest including LV mechanics.

	General Population	CFVR < 2.0	CFVR ≥ 2.0	
	*n* = 130	*n* = 35	*n* = 95	*p*
HR (bpm)	67 ± 13	68 ± 13	66 ± 12	0.482
SBP (mmHg)	131 ± 22	141 ± 26	127 ± 19	0.002
DBP (mmHg)	71 ± 15	77 ± 19	69 ± 13	0.005
iLVEDV (ml/m2)	55 ± 16	57 ± 22	54 ± 14	0.403
iLVESV (ml/m2)	21.7 ± 9.8	23 ± 13	31 ± 8	0.206
LVEF 3D (%)	61 ± 8	59 ± 9	61 ± 7	0.458
LV force index (mmHg/mL/m^2^)	6.7 (5.2–7.9)	6.9 (5.1–9.2)	6.6 (5.1–7.7)	0.332
B lines	0 (0–5)	0 (0–10)	0 (0–5)	0.251
GLS (%)	20.2 ± 3.3	20.1 ± 3.8	20.2 ± 3	0.971
Epicardial LS (%)	17.9 ± 3	17.9 ± 2	17.9 ± 3	0.915
Mesocardial LS (%)	20.3 ± 3.3	20 ± 3.7	20.4 ± 3.2	0.526
Endocardial LS (%)	23.5 ± 3.8	23.5 ± 4.6	23.3 ± 4.6	0.746
Delta endo-epi (%)	5.3 ± 1.6	5.6 ± 2	5.4 ± 1.4	0.557
MD (ms)	49.5 ± 15.5	53.8 ± 18	47.9 ± 14.3	0.056

CFVR: coronary flow velocity reserve. HR: heart rate, BPM: beats per minute, SBP: systolic blood pressure, DBP: diastolic blood pressure, iLVEDV: indexed left ventricular end-diastolic volume, iLVESV: indexed left ventricular end-systolic volume, LVEF: left ventricular ejection fraction, LV: left ventricle, GLS: global longitudinal strain, LS: longitudinal strain, MD: mechanical dispersion. All values are expressed as mean and SD, except B-lines (median and IQR).

**Table 4:** Clinical and echocardiographic characteristics at stress including LV mechanics.

	General Population	CFVR < 2.0	CFVR ≥ 2.0	
	*n* = 130	*n* = 35	*n* = 95	*p*
HR (bpm)	85 ± 14	82 ± 13	86 ± 14	0.183
SBP (mmHg)	129 ± 20	137 ± 18	125 ± 20	0.002
DBP (mmHg)	68 ± 14	70 ± 14	67 ± 14	0.397
iLVEDV (ml/m^2^)	56.1 ± 16	56.9 ± 19	55.8 ± 15	0.735
iLVESV (ml/m^2^)	17 ± 8.7	18.7 ± 12	16.3 ± 6.7	0.166
LVEF 3D (%)	70 ± 9	68 ± 10	70 ± 8	0.301
LV force index (mmHg/mL/m^2^)	8.2 (6.4–10.8)	8.6 (6.2–11.8)	8.2 (6.5–10.3)	0.653
B lines	0 (0–13)	1 (0–13)	0 (0–7)	0.199
GLS (%)	22.9 ± 3.8	21 ± 4.5	23.6 ± 3.3	0.007
MD (ms)	45.2 ± 17.7	53.9 ± 15	41.9 ± 17.6	0.0005
Epicardial LS (%)	20.1 ± 3.5	18.2 ± 4	20.7 ± 3.5	0.0002
Mesocardial LS (%)	23 ± 4.1	20.9 ± 4.9	23.8 ± 3.5	0.0004
Endocardial LS (%)	26.3 ± 4.7	24.1 ± 5.4	27.1 ± 3.6	0.001
Delta endo-epi	6.2 ± 2.2	5.9 ± 2.3	6.4 ± 2.1	0.262
LV contractile reserve	1.33 ± 0.42	1.33 ± 0.4	1.33 ± 0.43	0.844
LV Synchrony Delta	6 (4.7–16)	3 (−8–8.1)	7 (−1–18.8)	0.035
Strain Stress-Rest Delta	2.9 (0.6–4.8)	0.4 (−2–3.4)	3.3 (1–3–5.3)	<0.0001
Strain Reserve	1.14 ± 0.16	1.01 (0.91–1.16)	1.17 (1.06–1.28)	<0.0001
Endo-Epi Reserve	1.13 (0.94–1.45)	1.0 (0.89–1.32)	1.2 (0.96–1.47)	0.047
HR delta	18 (10–26)	13 (7–23)	19(11–29)	0.041
HRR	1.27 (1.13–1.45)	1.24 (1.11–1.38)	1.29 (1.17–1.47)	0.044

CFVR: coronary flow velocity reserve. HR: heart rate, BPM: beats per minute, SBP systolic blood pressure, DBP: diastolic blood pressure, iLVEDV: indexed left ventricular end-diastolic volume, iLVESV: indexed left ventricular end-systolic volume, LVEF: left ventricular ejection fraction, LV: left ventricle, GLS: global longitudinal strain, MD: mechanical dispersion, LS: longitudinal strain, HRR: heart rate reserve. All values are expressed as mean and SD, except B-lines (median and IQR).

## Data Availability

The data presented in this study are available on request from the corresponding author.

## References

[1] Merz C.N.B., Pepine C.J., Walsh M.N., Fleg J.L., Camici P.G., Chilian W.M., Clayton J.A., Cooper L.S., Crea F., Di Carli M. (2017). Ischemia and No Obstructive Coronary Artery Disease (INOCA). Circulation.

[2] Jespersen L., Hvelplund A., Abildstrøm S.Z., Pedersen F., Galatius S., Madsen J.K., Jørgensen E., Kelbaek H., Prescott E. (2011). Stable angina pectoris with no obstructive coronary artery disease is associated with increased risks of major adverse cardiovascular events. Eur. Heart J..

[3] Gulati M., Cooper-DeHoff R.M., McClure C., Johnson B.D., Shaw L.J., Handberg E., Zineh I., Kelsey S.F., Arnsdorf M.F., Black H.R. (2009). Adverse Cardiovascular Outcomes in Women with Nonobstructive Coronary Artery Disease. Arch. Intern. Med..

[4] Sara J.D., Widmer R.J., Matsuzawa Y., Lennon R.J., Lerman L.O., Lerman A. (2015). Prevalence of Coronary Microvascular Dysfunction Among Patients with Chest Pain and Nonobstructive Coronary Artery Disease. JACC Cardiovasc. Interv..

[5] Sucato V., Novo G., Saladino A., Evola S., Galassi A.R. (2020). Coronary microvascular dysfunction. Minerva Cardioangiol..

[6] Ciampi Q., Zagatina A., Cortigiani L., Gaibazzi N., Daros C.B., Zhuravskaya N., Wierzbowska-Drabik K., Kasprzak J.D., de Castro e Silva Pretto J.L., D’Andrea A. (2019). Functional, Anatomical, and Prognostic Correlates of Coronary Flow Velocity Reserve During Stress Echocardiography. J. Am. Coll. Cardiol..

[7] Picano E., Pellikka P.A. (2014). Stress echo applications beyond coronary artery disease. Eur. Heart J..

[8] Knuuti J., Wijns W., Saraste A., Capodanno D., Barbato E., Funck-Brentano C., Prescott E., Storey R.F., Deaton C., Cuisset T. (2020). 2019 ESC Guidelines for the diagnosis and management of chronic coronary syndromes: The Task Force for the diagnosis and management of chronic coronary syndromes of the European Society of Cardiology (ESC). Eur. Heart J..

[9] Kunadian V., Chieffo A., Camici P.G., Berry C., Escaned J., Maas A.H.E.M., Prescott E., Karam N., Appelman Y., Fraccaro C. (2020). An EAPCI Expert Consensus Document on Ischaemia with Non-Obstructive Coronary Arteries in Collaboration with European Society of Cardiology Working Group on Coronary Pathophysiology & Microcirculation Endorsed by Coronary Vasomotor Disorders International Study Group. Eur. Heart J..

[10] Lang R.M., Badano L.P., Mor-Avi V., Afilalo J., Armstrong A., Ernande L., Flachskampf F.A., Foster E., Goldstein S.A., Kuznetsova T. (2015). Recommendations for Cardiac Chamber Quantification by Echocardiography in Adults: An Update from the American Society of Echocardiography and the European Association of Cardiovascular Imaging. Eur. Heart J. Cardiovasc. Imaging.

[11] Sicari R., Nihoyannopoulos P., Evangelista A., Kasprzak J., Lancellotti P., Poldermans D., Voigt J.-U., Zamorano J.L., on behalf of the European Association of Echocardiography (2008). Stress Echocardiography Expert Consensus Statement—Executive Summary: European Association of Echocardiography (EAE) (a registered branch of the ESC). Eur. Heart J..

[12] Pellikka P.A., Arruda-Olson A., Chaudhry F.A., Chen M.H., Marshall J.E., Porter T.R., Sawada S.G. (2020). Guidelines for Performance, Interpretation, and Application of Stress Echocardiography in Ischemic Heart Disease: From the American Society of Echocardiography. J. Am. Soc. Echocardiogr..

[13] Scali M.C., Zagatina A., Simova I., Zhuravskaya N., Ciampi Q., Paterni M., Marzilli M., Carpeggiani C., Picano E., Citro R. (2017). B-lines with Lung Ultrasound: The Optimal Scan Technique at Rest and During Stress. Ultrasound Med. Biol..

[14] Scali M.C., Zagatina A., Ciampi Q., Cortigiani L., D’Andrea A., Daros C.B., Zhuravskaya N., Kasprzak J.D., Wierzbowska-Drabik K., Pretto J.L.D.C.E.S. (2020). Lung Ultrasound and Pulmonary Congestion During Stress Echocardiography. JACC Cardiovasc. Imaging.

[15] Cortigiani L., Huqi A., Ciampi Q., Bombardini T., Bovenzi F., Picano E. (2018). Integration of Wall Motion, Coronary Flow Velocity, and Left Ventricular Contractile Reserve in a Single Test: Prognostic Value of Vasodilator Stress Echocardiography in Patients with Diabetes. J. Am. Soc. Echocardiogr..

[16] Ciampi Q., Zagatina A., Cortigiani L., Wierzbowska-Drabik K., Kasprzak J.D., Haberka M., Djordjevic-Dikic A., Beleslin B., Boshchenko A., Ryabova T. (2021). Prognostic value of stress echocardiography assessed by the ABCDE protocol. Eur. Heart J..

[17] Ciampi Q., Picano E., Paterni M., Daros C.B., Simova I., Pretto J.L.D.C.E.S., Scali M.C., Gaibazzi N., Severino S., Djordjevic-Dikic A. (2017). Quality control of regional wall motion analysis in stress Echo 2020. Int. J. Cardiol..

[18] Scali M.C., Ciampi Q., Picano E., Bossone E., Ferrara F., Citro R., Colonna P., Costantino M.F., Cortigiani L., Andrea A.D. (2018). Quality control of B-lines analysis in stress Echo 2020. Cardiovasc. Ultrasound.

[19] Carpeggiani C., Ciampi Q., Paterni M., De Nes M., Zagatina A., Simova I., Djordievic-Dikic A., Citro R., Colonna P., Picano E. (2018). Multi-step Web-based Training: The Road to Stress Echo 2020. Rev. Argent. Cardiol..

[20] Mada R.O., Lysyansky P., Daraban A.M., Duchenne J., Voigt J.-U. (2015). How to Define End-Diastole and End-Systole?. JACC Cardiovasc. Imaging.

[21] Cortigiani L., Carpeggiani C., Landi P., Raciti M., Bovenzi F., Picano E. (2019). Usefulness of Blunted Heart Rate Reserve as an Imaging-Independent Prognostic Predictor During Dipyridamole Stress Echocardiography. Am. J. Cardiol..

[22] Hearse D.J. (1994). Myocardial ischaemia: Can we agree on a definition for the 21st century?. Cardiovasc. Res..

[23] Ross J. (1986). Assessment of ischemic regional myocardial dysfunction and its reversibility. Circulation.

[24] Hanekom L., Cho G.-Y., Leano R., Jeffriess L., Marwick T.H. (2007). Comparison of two-dimensional speckle and tissue Doppler strain measurement during dobutamine stress echocardiography: An angiographic correlation. Eur. Heart J..

[25] Ng A., Sitges M., Pham P.N., Tran D.T., Delgado V., Bertini M., Nucifora G., Vidaic J., Allman C., Holman E.R. (2009). Incremental value of 2-dimensional speckle tracking strain imaging to wall motion analysis for detection of coronary artery disease in patients undergoing dobutamine stress echocardiography. Am. Heart J..

[26] Aggeli C., Lagoudakou S., Felekos I., Panagopoulou V., Kastellanos S., Toutouzas K., Roussakis G., Tousoulis D. (2015). Two-dimensional speckle tracking for the assessment of coronary artery disease during dobutamine stress echo: Clinical tool or merely research method. Cardiovasc. Ultrasound.

[27] Yü Y., Villarraga H.R., Saleh H.K., Cha S.S., Pellikka P.A. (2012). Can ischemia and dyssynchrony be detected during early stages of dobutamine stress echocardiography by 2-dimensional speckle tracking echocardiography?. Int. J. Cardiovasc. Imaging.

[28] Chattopadhyay S., Alamgir M.F., Nikitin N.P., Fraser A.G., Clark A.L., Cleland J.G. (2008). The effect of pharmacological stress on intraventricular dyssynchrony in left ventricular systolic dysfunction. Eur. J. Heart Fail..

[29] Michelsen M.M., Pena A., Mygind N.D., Bech J., Gustafsson I., Kastrup J., Hansen H.S., Høst N., Hansen P.R., Prescott E. (2018). Coronary microvascular dysfunction and myocardial contractile reserve in women with angina and no obstructive coronary artery disease. Echocardiography.

[30] Ikonomidis I., Tzortzis S., Paraskevaidis I., Triantafyllidi H., Papadopoulos C., Papadakis I., Trivilou P., Parissis J., Anastasiou-Nana M., Lekakis J. (2012). Association of abnormal coronary microcirculatory function with impaired response of longitudinal left ventricular function during adenosine stress echocardiography in untreated hypertensive patients. Eur. Heart J. Cardiovasc. Imaging.

[31] Lafitte S., Bordachar P., Lafitte M., Garrigue S., Reuter S., Reant P., Serri K., Lebouffos V., Berrhouet M., Jais P. (2006). Dynamic Ventricular Dyssynchrony: An Exercise-Echocardiography Study. J. Am. Coll. Cardiol..

[32] Rodríguez-Zanella H., Haugaa K., Boccalini F., Secco E., Edvardsen T., Badano L.P., Muraru D. (2018). Physiological Determinants of Left Ventricular Mechanical Dispersion. JACC: Cardiovasc. Imaging.

[33] Schnell F., Matelot D., Daudin M., Kervio G., Mabo P., Carré F., Donal E. (2017). Mechanical Dispersion by Strain Echocardiography: A Novel Tool to Diagnose Hypertrophic Cardiomyopathy in Athletes. J. Am. Soc. Echocardiogr..

[34] Tzortzis S., Ikonomidis I., Triantafyllidi H., Trivilou P., Pavlidis G., Katsanos S., Katogiannis K., Birba D., Thymis J., Makavos G. (2020). Optimal Blood Pressure Control Improves Left Ventricular Torsional Deformation and Vascular Function in Newly Diagnosed Hypertensives: A 3-Year Follow-up Study. J. Cardiovasc. Transl. Res..

[35] Leung M.C.H., Meredith I.T., Cameron J. (2006). Aortic stiffness affects the coronary blood flow response to percutaneous coronary intervention. Am. J. Physiol. Circ. Physiol..

[36] Sicari R., Rigo F., Cortigiani L., Gherardi S., Galderisi M., Picano E. (2009). Additive Prognostic Value of Coronary Flow Reserve in Patients with Chest Pain Syndrome and Normal or Near-Normal Coronary Arteries. Am. J. Cardiol..

